# Blood Plasma Proteomic Profiling of Common Carp (*Cyprinus carpio*) Exposed to Glyphosate, AMPA, and Their Mixture

**DOI:** 10.3390/jox16030085

**Published:** 2026-05-16

**Authors:** Victoria Yurchenko, Alexey Morozov

**Affiliations:** Papanin Institute for Biology of Inland Waters, Russian Academy of Sciences, Borok 152742, Russia

**Keywords:** glyphosate, AMPA, herbicide, mixture exposure, common carp, blood plasma proteomics, label-free quantification, immune-related proteins, aquatic ecotoxicology

## Abstract

Glyphosate and its primary metabolite aminomethylphosphonic acid (AMPA) are widely detected in aquatic environments, yet their combined effects on fish remain insufficiently understood. This study used label-free blood plasma proteomic profiling to explore molecular patterns associated with 14-day exposure of juvenile common carp (*Cyprinus carpio*) to environmentally relevant concentrations of glyphosate (100 µg/L), AMPA (100 µg/L), and their mixture (50 + 50 µg/L). Across the three exposure groups, 41 proteins of interest showed pronounced abundance differences relative to the control based on fold-change selection criteria. These proteins were mainly associated with immune recognition, innate immune and complement-associated functions, coagulation and extracellular protease regulation, lipid/sterol transport, and extracellular matrix organization. In the mixture group, proteins of interest spanned several functional categories, suggesting that combined exposure deserves further attention in future studies of plasma-level responses to glyphosate and AMPA. Overall, these findings provide preliminary insights into blood plasma protein patterns associated with systemic responses of fish to glyphosate, AMPA, and their mixture at environmentally relevant concentrations and highlight the importance of considering parent compounds, metabolites, and their co-occurrence when assessing the potential biological effects of herbicide contamination in aquatic ecosystems.

## 1. Introduction

Fish are a crucial component of global food and nutrition security, providing an important source of protein and essential nutrients. Water pollution poses a significant threat to fish health, with herbicides and their degradation products being among the contaminants of concern. By 2022, global agricultural pesticide use had doubled compared to 1990 levels, with herbicides increasing their share from 40% to 50% of total pesticide use during this period [1].

Glyphosate [N-(phosphonomethyl)glycine] is among the most widely used herbicides worldwide because of its broad-spectrum efficacy and compatibility with genetically modified, glyphosate-resistant crops [2,3,4]. A major metabolite resulting from microbial degradation of glyphosate in soil is (aminomethyl)phosphonic acid (AMPA), which often exhibits greater environmental persistence than its parent compound [2,5]. Both glyphosate and AMPA can enter aquatic ecosystems through leaching and runoff following agricultural or urban applications [6,7], as well as via off-site airborne transport with dust [8]. Furthermore, AMPA can be formed in surface waters through the photodegradation of aminopolyphosphonates, which are widely used as antiscalants in various industrial and domestic applications [6]. In addition, a recent study identified municipal wastewater as a significant source of glyphosate in European rivers and suggested that aminopolyphosphonates may act as a common precursor for both AMPA and glyphosate in aquatic environments [9]. Thus, accumulating evidence indicates that the dispersal of glyphosate and AMPA can result in long-range contamination within watersheds and along river systems [5].

Glyphosate and AMPA are detected in surface waters worldwide [5], usually at relatively low concentrations, reaching tenths of a microgram or several micrograms per liter [9,10]. However, in regions with intensive herbicide use, contamination levels can be substantially higher [11,12,13], exceeding the hundred-microgram threshold—a regulatory acceptable concentration proposed by European authorities (RAC = 100 µg a.e./L) [3].

There is evidence that glyphosate and AMPA at environmentally relevant concentrations may affect fish health by dysregulating multiple cellular pathways, including oxidative phosphorylation, necroptosis, and apoptosis [14]. These molecular disturbances are associated with cellular stress responses, such as dysregulation of antioxidant and biotransformation enzymes [15,16,17,18,19], inhibition of membrane-bound ATPases [20], as well as reproductive and developmental toxicity [20,21]. Furthermore, studies on zebrafish have shown that glyphosate and AMPA at relatively low levels can disturb immune-related pathways in fish liver [14,16,19].

Building upon this evidence, we hypothesized that exposure to glyphosate and AMPA may be associated with changes in immune- and stress-related blood plasma proteins in fish. To explore this possibility, we used juvenile common carp (*Cyprinus carpio*) as a relevant fish species for investigating biological effects of environmental contaminants. The aim of this work was to perform an exploratory label-free proteomic profiling of blood plasma from common carp exposed to glyphosate, AMPA, and their mixture at environmentally relevant concentrations, with a focus on characterizing protein patterns associated with potential sublethal molecular responses.

## 2. Materials and Methods

### 2.1. Fish and Experimental Design

All experimental procedures were conducted in accordance with internationally accepted guidelines for the use of animals in research [22,23]. The study protocol was reviewed and approved by the Bioethics Committee of the Papanin Institute for Biology of Inland Waters, Russian Academy of Sciences (Protocol No. 9, 21 April 2023).

Juvenile common carp (*Cyprinus carpio*) were obtained from the “Sunoga” Field Experimental Station of the Papanin Institute for Biology of Inland Waters Russian Academy of Sciences. At the beginning of the experiment, fish were 4–5 months old.

Before exposure, fish were acclimated under laboratory conditions. A batch of carp was transferred to the experimental room and placed in two 45 L aquaria. After 28 days, fish were randomly distributed into 6 L experimental tanks and maintained for an additional 14 days before exposure. Each tank contained 5 L of water and five fish. During acclimation and exposure, fish were maintained under controlled conditions: 16 h light:8 h dark photoperiod, water temperature 21.9 ± 0.3 °C, pH 8.0 ± 0.1, dissolved oxygen 7.4 ± 0.3 mg/L. Fish were fed ad libitum twice daily with a commercial complete feed (TetraMin XL Granules; crude protein 48%, crude fat 7%, crude fiber 2%; granule size 2–3 mm).

To maintain stable exposure conditions, 50% of the water volume was renewed every second day. This interval was selected based on the reported persistence of both compounds, with half-lives in aquatic systems exceeding several days [6,7]. Thus, renewal every 48 h was considered sufficient to minimize concentration decline due to degradation while avoiding excessive handling stress.

To assess the effects of glyphosate and AMPA, the following treatment groups were established: control water without added contaminants, 100 µg/L glyphosate, 100 µg/L AMPA, and a mixture of 50 µg/L glyphosate + 50 µg/L AMPA. The exposure lasted 14 days and was performed in triplicate (three tanks per group and five fish per tank). Stock solutions were prepared in distilled water using analytical-grade reagents: N-(Phosphonomethyl)glycine (96%, Sigma-Aldrich, Saint Louis, MO, USA, product no. 337757) and (Aminomethyl)phosphonic acid (99%, Sigma-Aldrich, product no. 324817).

### 2.2. Sampling and Plasma Pooling

At the end of the experiment, fish were anesthetized by immersion in a 500 mg/L MS-222 solution until loss of equilibrium, which occurred within approximately 30 s. Fish were then removed from the anesthetic solution, weighed, and blood was collected from the caudal vessels. The same procedure was applied to all experimental groups to minimize procedure-related variability. The average body mass of fish at sampling was 2.18 ± 0.35 g.

**Figure 1:**
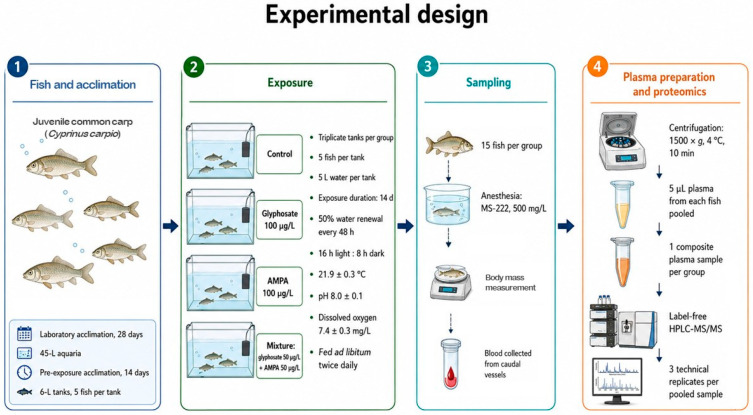
Schematic overview of the experimental design. The workflow includes acclimation, exposure, blood sampling, plasma pooling, and label-free HPLC-MS/MS analysis.

Blood samples (20 µL) were collected into 200 μL centrifuge tubes containing 10 µL of heparin solution (1250 U/mL in PBS). To obtain plasma, blood samples were centrifuged at 1500× *g* and 4 °C for 10 min. Due to the small body size of juvenile fish and the limited volume of plasma obtained from each individual, plasma samples were pooled within each treatment group to obtain sufficient material for HPLC-MS/MS analysis. For each group, 5 µL blood plasma aliquots from all 15 fish were combined to generate one pooled plasma sample. The resulting samples were stored in liquid nitrogen until further proteomic analysis. A schematic overview of the experimental design is given in Figure 1.

### 2.3. Protein Digestion and Label-Free HPLC–MS/MS Analysis

Proteomic analysis was carried out at the “Human Proteome” Core Facility (IBMC, Moscow, Russia). Each pooled plasma sample was analyzed in three technical HPLC-MS/MS replicates to obtain more robust protein abundance estimates. Protein concentration in the blood plasma samples was determined using the bicinchoninic acid colorimetric assay according to the manufacturer’s instructions (Pierce, Rockford, IL, USA). Aqueous solutions of bovine serum albumin were used for calibration, and total protein concentrations were measured using a NanoDrop ND-1000 spectrophotometer (Thermo Fisher Scientific, Wilmington, DE, USA) at a wavelength of 562 nm.

To perform protein digestion, plasma volumes corresponding to 100 µg of total protein per sample were diluted six-fold with 50 mM triethylammonium bicarbonate (TEAB) buffer. For reduction and alkylation of disulfide bonds, samples were supplemented with 2 µL of 0.5 M tris(2-carboxyethyl)phosphine (TCEP; Thermo Fisher Scientific) and 4 µL of 400 mM chloroacetamide (CAA; Sigma-Aldrich), followed by thorough mixing. The samples were incubated at 80 °C for 40 min and subsequently cooled to room temperature.

Protein digestion was performed by adding 70 µL of 50 mM TEAB and sequencing-grade trypsin (Promega, Walldorf, Germany) at a concentration of 0.2 µg/µL, at an enzyme-to-protein ratio of 1:50 (*w*/*w*), corresponding to 10 µL of trypsin solution per sample. The samples were incubated overnight. After digestion, formic acid was added to a final concentration of 5%, followed by centrifugation at 14,000× *g* for 20 min. The supernatants were dried in a Concentrator 5301 vacuum centrifuge (Eppendorf, Hamburg, Germany) at 45 °C. After complete drying, the samples were reconstituted in 100 µL of HPLC-grade water for subsequent peptide quantification using the colorimetric Peptide Assay kit (Thermo Fisher Scientific), according to the manufacturer’s instructions. The resulting peptide digests were dried in a vacuum concentrator and reconstituted in 0.1% formic acid to a final concentration of 2 µg/µL.

Label-free HPLC-MS/MS analysis of peptides was performed using an Ultimate 3000 RSLCnano UHPLC system (Thermo Fisher Scientific) coupled to a Q Exactive HF-X mass spectrometer (Thermo Fisher Scientific). One microgram of the peptide mixture was loaded onto an Acclaim μ-Precolumn (0.5 mm × 3 mm, 5 μm particle size; Thermo Fisher Scientific) at a flow rate of 10 µL/min for 4 min under isocratic conditions using buffer C (2% acetonitrile, 0.1% formic acid in deionized water) as the mobile phase. Then, peptides were separated on a PeakyEfficiency analytical column (FE, 100 μm × 30 cm, 1.9 μm particle size; Molecta, Moscow, Russia) using a gradient elution program with mobile phase A (0.1% formic acid in water) and mobile phase B (80% acetonitrile, 0.1% formic acid in water) at a flow rate of 0.3 µL/min. The column was initially equilibrated with 2% mobile phase B for 4 min, followed by a linear increase to 35% B over 65 min. The concentration of mobile phase B was then increased linearly to 99% over 6 min and maintained at this level for 10 min. Subsequently, mobile phase B was returned to 2% over 5 min. The total run time was 90 min.

Mass spectrometric analysis was performed in positive ionization mode using a nano-electrospray ionization (nESI) source (Thermo Fisher Scientific). The following instrumental parameters were applied: spray voltage 2.1 kV; capillary temperature 240 °C. Full MS scans were acquired over an *m*/*z* range of 300–1500 at a resolution of 120,000. For tandem mass spectrometry (MS/MS), spectra were acquired at a resolution of 15,000 over an *m*/*z* range from 100 to an automatically determined upper limit based on the precursor mass, with a maximum of 2000 *m*/*z*. Precursor ions were isolated using a ±1 Da isolation window. The maximum number of precursors selected for MS/MS per cycle was set to 40. The intensity threshold for precursor selection was 50,000, and the normalized collision energy (NCE) was set to 29. Only ions with charge states from z = 2+ to z = 6+ were selected for fragmentation. The maximum injection time was set to 50 ms for precursor ions and 110 ms for fragment ions. The automatic gain control (AGC) target values were set to 1 × 10^6^ for MS and 2 × 10^5^ for MS/MS. Dynamic exclusion was applied with an exclusion duration of 20 s.

Protein identification was performed using MaxQuant software (version 2.0.3.0) with the integrated Andromeda search engine. MS/MS spectra were searched against the UniProt proteome database (UP001108240 reference proteome). Trypsin was specified as the proteolytic enzyme, allowing up to one missed cleavage. The precursor mass tolerance was set to ±4.5 ppm, and the fragment mass tolerance was set to ±20 ppm. Oxidation of methionine and N-terminal protein acetylation were specified as variable modifications, whereas carbamidomethylation of cysteine was set as a fixed modification. Peptide-spectrum matches (PSMs), peptide identifications, and protein identifications were validated using a false discovery rate threshold of 1%. Label-free quantification was performed using the MaxLFQ algorithm [24].

### 2.4. Proteomic Data Analysis and Functional Annotation

In the primary dataset, contaminant proteins were removed, and proteins identified by at least two peptides were retained for further analysis. Raw proteomic data processing followed the general workflow described by Aguilan et al. [25]. LFQ intensities of the retained proteins were log-transformed and normalized by mean and slope. Missing values were imputed using a probabilistic minimum approach. Because the proteomic dataset did not include independent biological replicates, *p*-value-based testing of protein abundance differences was not applied.

Analytical reproducibility of technical HPLC-MS/MS replicates was assessed using pairwise Pearson correlation analysis and principal component analysis based on processed log_2_ LFQ intensities. Pairwise Pearson correlation coefficients among technical replicates ranged from 0.921 to 0.965, and principal component analysis showed close clustering of replicates within each pooled sample (Table S1). Therefore, all technical replicates were retained and used to calculate average protein abundance values for each sample.

Because the proteomic analysis was performed using one pooled sample per treatment group, protein abundance differences between each treatment group and the control were interpreted descriptively based on log_2_ fold-change values (log_2_FC). To reduce the inclusion of sporadically detected proteins while retaining proteins consistently detected in either the control or treatment, proteins were considered in treatment-to-control comparisons only if they were detected before imputation in at least two of three technical replicates in the control and/or treatment sample.

Proteins showing |log_2_FC| ≥ 1, corresponding to at least a two-fold abundance difference relative to the control, were selected as proteins of interest for exploratory functional interpretation. For visualization of abundance patterns, proteins that showed |log_2_FC| ≥ 1 in at least one treatment group were used to generate a descriptive heatmap using the SRplot online platform [26]. Proteins were hierarchically clustered by rows using a correlation-based distance metric and the complete linkage method. Row-wise scaling (z-score) was not applied, allowing color intensity to reflect the direction and magnitude of abundance differences relative to the control. The identification of shared and treatment-specific proteins of interest was performed using the Venny 2.1 online platform [27].

Functional interpretations of the log_2_FC-selected proteins were inferred from domain architecture derived from InterPro and zebrafish orthology provided by Ensembl; for proteins lacking high-confidence orthologues, functional assignments were based on conserved domain structure. Because the proteomic analysis was based on pooled samples, functional patterns were interpreted as hypothesis-generating.

## 3. Results

**Figure 2:**
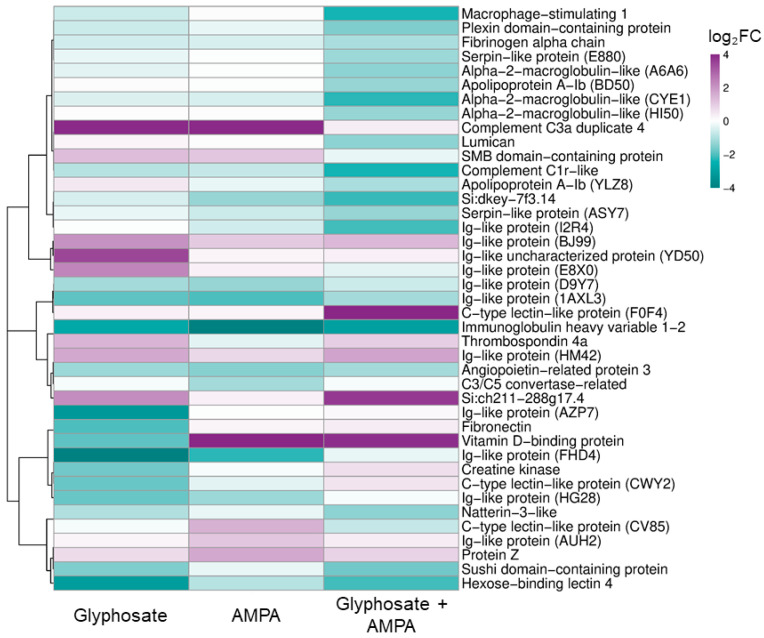
Heatmap of fold-change-selected blood plasma proteins in juvenile common carp exposed to glyphosate, aminomethylphosphonic acid (AMPA), and their mixture. The heatmap shows relative abundance differences for 41 proteins that met the detection and fold-change selection criteria. Proteins were hierarchically clustered by rows; the color scale was limited to −4 to +4 log_2_FC for visualization purposes. Full UniProt identifiers and log_2_FC data are provided in Table S2.

Application of the fold-change selection criteria resulted in 21 proteins of interest in the glyphosate group, 15 proteins in the AMPA group, and 25 proteins in the glyphosate + AMPA mixture group. Across all exposure groups, 41 unique proteins met the selection criteria in at least one group and were used for visualization (Figure 2).

Functional annotations of proteins with pronounced abundance differences were based on conserved domains and zebrafish orthology where available. In the glyphosate group, proteins of interest included several Ig-like domain-containing proteins, immunoglobulin heavy variable 1-2, hexose-binding lectin 4, a C-type lectin domain-containing protein, complement component C3a duplicate 4, a sushi domain-containing protein, fibronectin, thrombospondin 4a, vitamin D-binding protein, angiopoietin-related protein 3, creatine kinase, and an SMB domain-containing protein (Table S3). Functionally, these proteins were mainly related to immune recognition, innate immune and complement-associated processes, extracellular matrix organization, lipid/sterol transport, and energy metabolism.

In the AMPA group, proteins with pronounced abundance differences included several Ig-like domain-containing proteins, immunoglobulin heavy variable 1-2, a C-type lectin domain-containing protein, C3/C5 convertase, complement component C3a duplicate 4, protein Z-like vitamin K-dependent plasma glycoprotein, vitamin D-binding protein, angiopoietin-related protein 3, an apolipoprotein A-I domain-containing protein, and an SMB domain-containing protein (Table S4). These proteins were mainly associated with immune recognition and antigen binding, innate immune and complement-associated processes, coagulation regulation, lipid/sterol transport, and extracellular enzymatic or signaling activity.

For the combined glyphosate + AMPA exposure, the proteins of interest comprised immunoglobulin heavy variable 1-2, several Ig-like domain-containing proteins, macrophage stimulating 1, complement subcomponent C1r, alpha-2-macroglobulin-like and serpin domain-containing proteins, hexose-binding lectin 4, a C-type lectin domain-containing protein, natterin-3-like protein, a sushi domain-containing protein, fibrinogen alpha chain, apolipoprotein A-Ib, vitamin D-binding protein, angiopoietin-related protein 3, lumican, plexin domain containing 2b, and thrombospondin 4a (Table S5). The corresponding annotations pointed primarily to immune recognition, innate immune and complement-associated processes, inflammatory response regulation, extracellular proteolysis and coagulation, lipid/sterol transport, and extracellular matrix organization.

Comparative analysis revealed both exposure-specific and shared protein abundance patterns among the treatments. Five proteins were shared by all three groups, including immunoglobulin heavy variable 1-2, two Ig-like domain-containing proteins, vitamin D-binding protein, and angiopoietin-related protein 3 (Table S6).

## 4. Discussion

The present study used label-free proteomic profiling of blood plasma as an exploratory approach to examine molecular patterns associated with exposure of juvenile common carp to glyphosate, AMPA, and their mixture. Within this framework, proteins showing the most pronounced abundance differences relative to the control were selected for functional interpretation. The results pointed to several biological processes that may be involved in systemic responses to these compounds, including immune recognition, innate immune and complement-associated functions, coagulation and extracellular protease regulation, lipid/sterol transport, and extracellular matrix organization.

A prominent feature of the fold-change-selected protein set was the presence of multiple proteins related to immune recognition and innate defense, including several Ig-like domain-containing proteins, immunoglobulin heavy variable 1-2, C-type lectin domain-containing proteins, hexose-binding lectin 4, natterin-3-like protein, and macrophage stimulating 1. Immunoglobulin-like domains are widespread in proteins involved in antigen recognition, cell adhesion, and immune regulation [28,29], and their repeated occurrence among the selected proteins may reflect changes in plasma components associated with immune recognition. C-type lectin domain-containing proteins and hexose-binding lectin 4 may also be relevant to carbohydrate recognition and innate immune defense, as lectins in teleost fish are involved in host–pathogen interactions and can function as pattern-recognition molecules recognizing pathogen-associated molecular patterns [30,31].

The presence of natterin-3-like protein further supports the possible involvement of defense-related mechanisms. Natterin-like proteins were originally described as fish venom components but are now recognized more broadly as proteins involved in innate immune defense and host–pathogen interactions [32,33]. Taken together, these observations suggest that immune-related plasma proteins may be sensitive components of the systemic response to glyphosate-, AMPA-, and mixture-related exposure. However, because several of these proteins have broad domain-based annotations rather than experimentally characterized functions in common carp, their specific roles require further validation.

Another group of fold-change-selected proteins was associated with complement-related processes, coagulation, and extracellular protease regulation. This group included complement component C3a duplicate 4, complement subcomponent C1r, C3/C5 convertase, a sushi domain-containing protein, alpha-2-macroglobulin-like proteins, serpin domain-containing proteins, fibrinogen alpha chain, and protein Z-like vitamin K-dependent plasma glycoprotein. These proteins belong to interconnected plasma-associated systems involved in innate defense, inflammatory regulation, proteolytic control, and hemostasis. The occurrence of complement-associated proteins among the fold-change-selected set is consistent with previous reports that glyphosate exposure can affect immune functions in fish, including complement activity and phagocytic responses [34]. Complement is a central component of teleost innate immunity and contributes to pathogen recognition, opsonization, inflammatory signaling, chemotaxis, and cell lysis [35,36]. Therefore, the presence of complement-related plasma proteins among the proteins of interest may reflect the involvement of systemic immune and inflammatory regulation in the response to glyphosate-, AMPA-, and mixture-related exposure.

Protease inhibitors and coagulation-related proteins may also be relevant in this context. Alpha-2-macroglobulin-like proteins and serpins are of particular interest because these protein families regulate extracellular proteolysis and are functionally connected with inflammatory and plasma cascade systems. Members of the alpha-2-macroglobulin family act as broad-spectrum protease inhibitors and can interact with cytokines and growth factors, thereby linking protease control with immune and inflammatory regulation [37]. Serpins represent a large class of serine protease inhibitors involved in the regulation of coagulation, fibrinolytic, and complement cascades [38,39]. The occurrence of these proteins among the proteins of interest, together with fibrinogen alpha chain and protein Z, which are associated with coagulation and hemostatic regulation [40,41,42], points to possible involvement of plasma cascade systems linking innate defense, inflammation, extracellular proteolysis, and vascular homeostasis.

Another group of proteins of interest was related to lipid/sterol transport and systemic carrier functions. This group included two apolipoprotein A-Ib proteins, vitamin D-binding protein, angiopoietin-related protein 3, and an SMB domain-containing protein with a possible link to extracellular enzymatic or lipid mediator-related activity. The occurrence of these proteins among the fold-change-selected set suggests that exposure to glyphosate, AMPA, and their mixture may be associated with changes in plasma transport and metabolic homeostasis. Apolipoproteins are major components of plasma lipoproteins and participate in lipid transport, cholesterol homeostasis, and immune-metabolic regulation; in particular, apolipoprotein A-I is known to exert anti-inflammatory effects by modulating immune cell functions [43]. Angiopoietin-related protein 3 is also involved in lipid metabolism and has been described as a regulator of triglyceride and lipoprotein metabolism [44,45]. Vitamin D-binding protein, although best known as a carrier of vitamin D metabolites, also belongs to the albumin family of plasma carrier proteins and may reflect broader changes in systemic transport functions. These observations are consistent with recent transcriptomic and functional evidence from zebrafish showing that glyphosate exposures can be associated with altered metabolic activity, lipid metabolism-related pathways, oxidative stress, and inflammatory responses in fish tissues [46]. However, in the present study, these proteins should be interpreted as indicators of possible changes in carrier and lipid-related functions rather than as direct evidence of metabolic pathway disruption.

Proteins related to extracellular matrix organization and cell adhesion were also represented among the proteins of interest. These included fibronectin, thrombospondin 4a, lumican, and a plexin domain-containing 2b protein. Fibronectin and thrombospondins are extracellular matrix-associated glycoproteins involved in cell adhesion, matrix organization, and tissue remodeling, while lumican is a small leucine-rich proteoglycan associated with collagen organization [47,48,49]. The presence of these proteins in the fold-change-selected plasma protein set may indicate that exposure-related systemic patterns involve extracellular matrix-associated or tissue remodeling-related processes [50]. In blood plasma, extracellular matrix-associated proteins should be interpreted cautiously, because their abundance may reflect several non-mutually exclusive processes, including tissue remodeling, vascular or endothelial responses, extracellular protein turnover, or release of extracellular components into circulation [51,52]. Nevertheless, their occurrence together with complement-, coagulation-, and protease-regulation-related proteins may point to coordinated involvement of extracellular and plasma cascade systems in the systemic response of fish to these contaminants.

The combined glyphosate + AMPA group contained the largest number of proteins meeting the fold-change selection criteria. This observation should be interpreted descriptively, because the pooled design does not allow statistical comparison of the extent of response among treatments. Nevertheless, the mixture-associated protein set included proteins from several functional categories, including immune recognition, complement and coagulation, extracellular protease regulation, lipid transport, and extracellular matrix organization. This pattern suggests that combined exposure may involve multiple plasma-associated processes and supports the need to consider glyphosate and AMPA together when assessing their potential biological effects in aquatic organisms. Because blood plasma integrates signals from multiple tissues and physiological systems, these protein patterns may be relevant to sublethal systemic responses, although their consequences for physiological performance, fitness, or population-level outcomes remain to be tested.

## 5. Limitations of the Study

The main limitation of the present study is the use of one pooled plasma sample per treatment group for proteomic analysis. Although each pooled sample represented plasma from 15 fish, pooling did not preserve biological replication at the HPLC-MS/MS level and therefore did not allow assessment of inter-individual or inter-tank variability. Consequently, no statistical testing of protein abundance differences was performed, and the proteomic results were interpreted descriptively based on fold-change patterns. Accordingly, the selected proteins should be considered proteins of interest for exploratory functional interpretation rather than statistically confirmed differentially abundant proteins. Another limitation is that functional interpretation relied largely on database annotations, conserved domain architecture, and zebrafish orthology. Because many common carp protein entries remain predicted or incompletely characterized, the specific functions of some proteins in carp blood plasma cannot be assigned with full confidence. For these reasons, the observed patterns should be regarded as hypothesis-generating and require confirmation in future studies.

## 6. Conclusions

This study used label-free blood plasma proteomic profiling to explore molecular patterns associated with exposure of juvenile common carp to glyphosate, AMPA, and their mixture at environmentally relevant concentrations. Using a fold-change-based descriptive approach, we selected proteins of interest that were mainly related to immune recognition, innate immune and complement-associated functions, coagulation and extracellular protease regulation, lipid/sterol transport, and extracellular matrix organization. Together, these patterns suggest that blood plasma proteomics can provide useful mechanistic insights into systemic responses of fish to herbicide-related contaminants. In this study, the mixture group contained the largest number of proteins meeting the fold-change selection criteria, supporting the relevance of combined exposure scenarios in aquatic ecotoxicology. Overall, these findings highlight the importance of considering not only glyphosate itself but also its metabolite AMPA and their co-occurrence when assessing the potential biological effects of herbicide contamination in aquatic ecosystems.

## Data Availability

The raw data supporting the conclusions of this article will be made available by the authors on request.

## Supplementary Materials

The following supporting information can be downloaded at: https://www.mdpi.com/article/10.3390/jox16030085/s1, Table S1: Analytical reproducibility of technical HPLC-MS/MS replicates assessed by pairwise Pearson correlation analysis; Table S2: Fold-change-selected blood plasma proteins used for heatmap visualization; Table S3: Domain- and orthology-based functional annotation of fold-change-selected proteins in the blood plasma of common carp exposed to glyphosate; Table S4: Domain- and orthology-based functional annotation of fold-change-selected proteins in the blood plasma of common carp exposed to AMPA; Table S5: Domain- and orthology-based functional annotation of fold-change-selected proteins in the blood plasma of common carp exposed to Glyphosate + AMPA; Table S6: Overlap of fold-change-selected blood plasma proteins among exposure groups.

## Abbreviations

The following abbreviations are used in this manuscript:

AMPA	(Aminomethyl)phosphonic acid
HPLC	High-performance liquid chromatography
LFQ	Label-free quantification
MS/MS	Tandem mass spectrometry
