# Supplementary material for: Blood Plasma Proteomic Profiling of Common Carp (Cyprinus carpio) Exposed to Glyphosate, AMPA, and Their Mixture
Source: J Xenobiot. 2026 May 16;16(3):85. doi: 10.3390/jox16030085 (PMC13214966; doi:10.3390/jox16030085)
Supplement: Supplementary file 1 [file jox-16-00085-s001.zip › Table S3.pdf]

Table S3. Domain- and orthology-based functional annotation of fold-change-selected proteins in the blood plasma of common carp exposed to glyphosate

UniProt ID	Protein name, UniProt	$Log_2(FC)$	Conserved domains, InterPro	Zebrafish orthologue, Ensembl	Orthology type, Ensembl	High confidence, Ensembl	Functional interpretation
A0A8C1FHD4	Ig-like domain-containing protein	-6.26	Immunoglobulin V-set domain	Not identified	–	–	Immune recognition*
A0A8C1DWU1	Ig-like domain-containing protein	-3.21	Immunoglobulin V-set domain	igl3v1	Many-to-many	No	Immune recognition*
A0A8C1EAX2	Hexose-binding lectin 4	-3.10	Collagen triple helix repeat (20 copies); 6-Phosphogluconate Dehydrogenase, domain 3; C-type lectin-like domain	hbl4	One-to-many	Yes	Carbohydrate binding and extracellular matrix interactions**
A0A8C1CNP4	Immunoglobulin heavy variable 1-2	-2.71	Immunoglobulin V-set domain	ighv1-2	One-to-many	Yes	Immune recognition, antigen binding**
A0A8C1CTH0	Fibronectin	-2.01	Fibronectin domains	fn1b	One-to-many	Yes	Extracellular matrix organization and cell adhesion**
A0A8C1AXL3	Ig-like domain-containing protein	-1.87	Immunoglobulin V-set domain	Not identified	–	–	Immune recognition*
A0A8C0XYD7	Vitamin D-binding protein	-1.82	Albumin domain profile	gc	One-to-many	Yes	Lipid/sterol transport and systemic carrier protein function**
A0A8C1HG28	Ig-like domain-containing protein	-1.79	Immunoglobulin V-set domain	Not identified	–	–	Immune recognition*
A0A8C1CWY2	C-type lectin domain-containing protein	-1.77	C-type lectin-like domain	Not identified	–	–	Carbohydrate recognition and innate immune-related processes*
A0A8C1I2R0	Creatine kinase	-1.72	Glutamine synthetase/guanido kinase domain	ckba	One-to-one	Yes	Energy metabolism, phosphagen system**
A0A8C1CXV8	Sushi domain-containing protein	-1.57	Sushi/CCP/SCR domains	Not identified	–	–	Complement-related regulation, immune-

							associated extracellular interactions*
A0A8C1A381	Angiopoietin-related protein 3	-1.20	Fibrinogen C-terminal domain profile	angptl3	One-to-many	Yes	Lipid metabolism and fibrinogen-domain-associated extracellular function**
A0A8C1D9Y7	Ig-like domain-containing protein	-1.09	Immunoglobulin V-set domain; Ig-like domain	si:ch73-34h11.1	Many-to-many	No	Immune recognition*
A0A8C1FLD2	SMB domain-containing protein	1.28	Somatomedin B-like domains; Alkaline phosphatase-like domain; DNA/RNA non-specific endonuclease domain	enpp2	One-to-one	No	Extracellular enzymatic/signaling activity; possible lipid mediator metabolism*
A0A8C1DPE5	Thrombospondin 4a	1.48	Thrombospondin N-terminal-like domains	thbs4a	One-to-many	Yes	Extracellular matrix organization and cell adhesion**
A0A8C1HM42	Ig-like domain-containing protein	1.66	Ig-like domain profile	ighv10-1	One-to-many	No	Immune recognition*
A0A8C1BJ99	Ig-like domain-containing protein	2.14	Immunoglobulin V-set domain	Not identified	–	–	Immune recognition*
A0A8C1HPR1	Si:ch211-288g17.4	2.15	Not identified	si:ch211-288g17.4	One-to-many	Yes	Function uncertain; orthology-supported but poorly characterized
A0A8C1E8X0	Ig-like domain-containing protein	2.34	Immunoglobulin V-set domain; Immunoglobulin C1-set domain	Not identified	–	–	Immune recognition*
A0A8C0YD50	Uncharacterized protein	3.46	Immunoglobulin V-set domain	Not identified	–	–	Immune recognition*
A0A8C1GZQ5	Complement component c3a, duplicate 4	3.99	Macroglobulin domains; Anaphylatoxin domain signature; Complement_C3_C4_C5-like domain	c3a.4	One-to-many	Yes	Complement activation, innate immune response**

Note. *domain-based prediction; **prediction supported by conserved orthology.
