# Supplementary material for: Blood Plasma Proteomic Profiling of Common Carp (Cyprinus carpio) Exposed to Glyphosate, AMPA, and Their Mixture
Source: J Xenobiot. 2026 May 16;16(3):85. doi: 10.3390/jox16030085 (PMC13214966; doi:10.3390/jox16030085)
Supplement: Supplementary file 1 [file jox-16-00085-s001.zip › Table S4.pdf]

Table S4. Domain- and orthology-based functional annotation of fold-change-selected proteins in the blood plasma of common carp exposed to AMPA

UniProt ID	Protein name, UniProt	Log_2(FC)	Conserved domains, InterPro	Zebrafish orthologue, Ensembl	Orthology type, Ensembl	High confidence, Ensembl	Functional interpretation
A0A8C1CNP4	Immunoglobulin heavy variable 1-2	-3.94	Immunoglobulin V-set domain	ighv1-2	One-to-many	Yes	Antigen binding and adaptive humoral immune response**
A0A8C1FHD4	Ig-like domain-containing protein	-2.27	Immunoglobulin V-set domain	Not identified	–	–	Immune recognition*
A0A8C1AXL3	Ig-like domain-containing protein	-2.03	Immunoglobulin V-set domain	Not identified	–	–	Immune recognition*
A0A8C1D9Y7	Ig-like domain-containing protein	-1.31	Immunoglobulin V-set domain; Ig-like domain	si:ch73-34h11.1	Many-to-many	No	Immune recognition*
A0A8C1A381	Angiopoietin-related protein 3	-1.41	Fibrinogen C-terminal domain profile	angptl3	One-to-many	Yes	Lipid metabolism and fibrinogen-domain-associated extracellular function**
A0A8C1BRV9	Si:dkey-7f3.14	-1.28	Apolipoprotein A-I domain	si:dkey-7f3.14	One-to-one	Yes	Cholesterol and phospholipid transport and regulation of lipid homeostasis**
A0A8C1HG28	Ig-like domain-containing protein	-1.20	Immunoglobulin V-set domain	Not identified	–	–	Immune recognition*
A0A8C1H7Z7	C3/C5 convertase	-1.13	Short complement-like repeat (SCR); von Willebrand factor type A domain; Trypsin	cfb	Many-to-many	Yes	Complement activation, innate immune response**
A0A8C1BJ99	Ig-like domain-containing protein	1.06	Immunoglobulin V-set domain	Not identified	–	–	Immune recognition*
A0A8C1AUH2	Ig-like domain-containing protein	1.10	Immunoglobulin V-set domain	ighv14-1	One-to-many	Yes	Antigen binding and adaptive humoral immune response**
A0A8C1FLD2	SMB domain-containing protein	1.10	Somatomedin B-like domains; Alkaline	enpp2	One-to-one	No	Extracellular enzymatic/signaling

			phosphatase-like domain; DNA/RNA non-specific endonuclease domain				activity; possible lipid mediator metabolism*
A0A8C1CV85	C-type lectin domain-containing protein	1.55	C-type lectin domain profile	Not identified	–	–	Carbohydrate recognition and innate immune-related processes*
A0A8C1BIY5	Protein Z, vitamin K-dependent plasma glycoprotein b	1.69	Vitamin K-dependent carboxylation domain; EGF-like domain profile; Trypsin	prozb	One-to-one	Yes	Blood coagulation regulation and vitamin K-dependent plasma protein function**
A0A8C1GZQ5	Complement component c3a, duplicate 4	3.95	Macroglobulin domains; Anaphylatoxin domain signature; Complement_C3_C4_C5-like domain	c3a.4	One-to-many	Yes	Complement activation, innate immune response**
A0A8C0XYD7	Vitamin D-binding protein	4.07	Albumin domain profile	gc	One-to-many	Yes	Lipid/sterol transport and systemic carrier protein function**

Note. *domain-based prediction; **prediction supported by conserved orthology.
