# Supplementary material for: Blood Plasma Proteomic Profiling of Common Carp (Cyprinus carpio) Exposed to Glyphosate, AMPA, and Their Mixture
Source: J Xenobiot. 2026 May 16;16(3):85. doi: 10.3390/jox16030085 (PMC13214966; doi:10.3390/jox16030085)
Supplement: Supplementary file 1 [file jox-16-00085-s001.zip › Table S5.pdf]

Table S5. Domain- and orthology-based functional annotation of fold-change-selected proteins in the blood plasma of common carp exposed to Glyphosate + AMPA

**UniProt ID**	**Protein name, UniProt**	**Log_2(FC)**	**Conserved domains, InterPro**	**Zebrafish orthologue, Ensembl**	**Orthology type, Ensembl**	**High confidence, Ensembl**	**Functional interpretation**
A0A8C1CNP4	Immunoglobulin heavy variable 1-2	-2.96	Immunoglobulin V-set domain	ighv1-2	One-to-many	Yes	Antigen binding and adaptive humoral immune response**
A0A8C1DRS2	Macrophage stimulating 1	-2.35	PAN/Apple domain; Kringle domains; Trypsin-like serine protease domain	mst1	One-to-many	Yes	Macrophage activation and regulation of inflammatory responses**
A0A8C1C4R0	Complement subcomponent C1r	-2.30	CUB domains; Calcium-binding EGF-like domain; Sushi/CCP/SCR domains; Trypsin-like serine protease domain	c1r	One-to-many	Yes	Classical complement pathway initiation**
A0A8C1CYE1	Alpha-2-macroglobulin-like	-2.30	Macroglobulin domains; Alpha-2-Macroglobulin domains	a2ml	Many-to-many	Yes	Regulation of proteolysis and innate immune defense**
A0A8C1BRV9	Si:dkey-7f3.14	-2.15	Apolipoprotein A-I domain	si:dkey-7f3.14	One-to-one	Yes	Cholesterol and phospholipid transport and regulation of lipid homeostasis**
A0A8C1I2R4	Ig-like domain-containing protein	-2.13	Immunoglobulin V-set domain	si:dkey-234i14.13	One-to-many	No	Immune recognition*
A0A8C1EAX2	Hexose-binding lectin 4	-2.10	Collagen triple helix repeat (20 copies); 6-Phosphogluconate Dehydrogenase, domain 3; C-type lectin-like domain	hbl4	One-to-many	Yes	Carbohydrate binding and extracellular matrix interactions**
A0A8C1CXV8	Sushi domain-containing protein	-1.66	Sushi/CCP/SCR domains	Not identified	–	–	Complement-related regulation, immune-associated

							extracellular interactions*
A0A8C1FU04	Plexin domain containing 2b	-1.57	Plexin repeat	plxdc2b	One-to-many	Yes	Cell adhesion, extracellular signaling and tissue remodeling**
A0A8C0YL03	Natterin-3-like	-1.37	Aerolysin-type β-barrel pore-forming domain (Natterin-like)	Not identified	–	–	Innate immune defense*
A0A8C1A0Y0	Lumican	-1.35	Leucine-rich repeats	lum	One-to-one	Yes	Extracellular matrix organization and collagen-associated processes**
A0A8C1A6A6	Alpha-2-macroglobulin-like	-1.34	Macroglobulin (MG2) domains; Alpha-2-macroglobulin family domains; Alpha-2-macroglobulin receptor-binding domain	si:dkey-105h12.2	Many-to-many	Yes	Regulation of extracellular proteolysis and innate immune defense**
A0A8C1ASY7	Serpin domain-containing protein	-1.33	Serpin family A (alpha-1-antitrypsin–like) domain	serpina1	Many-to-many	Yes	Serine protease inhibition and regulation of extracellular proteolysis**
A0A8C1BD50	Apolipoprotein A-Ib	-1.32	Apolipoprotein A-I domain	apoa1b	One-to-many	Yes	Lipid metabolism and reverse cholesterol transport**
A0A8C1HI50	Alpha-2-macroglobulin-like	-1.31	Alpha-2-macroglobulin bait region domain	a2ml	Many-to-many	Yes	Regulation of proteolysis and innate immune defense**
A0A8C1E880	Serpin domain-containing protein	-1.24	Serpin domain	serpinf2a	One-to-one	Yes	Serine protease inhibition and regulation of fibrinolysis**
A0A8C1A381	Angiopoietin-related protein 3	-1.14	Fibrinogen C-terminal domain profile	angptl3	One-to-many	Yes	Lipid metabolism and fibrinogen-domain-associated

							extracellular function**
A0A8C1AXL3	Ig-like domain-containing protein	-1.14	Immunoglobulin V-set domain	Not identified	–	–	Immune recognition*
A0A8C0YLZ8	Apolipoprotein A-Ib	-1.07	Apolipoprotein A-I domain	apoa1b	One-to-many	Yes	Lipid metabolism and reverse cholesterol transport**
A0A8C1CTW8	Fibrinogen alpha chain	-1.05	Fibrinogen alpha/beta chain family domain; Fibrinogen beta and gamma chains, C-terminal globular domain	fga	One-to-one	Yes	Blood coagulation; fibrin clot formation; platelet aggregation**
A0A8C1BJ99	Ig-like domain-containing protein	1.38	Immunoglobulin V-set domain	Not identified	–	–	Immune recognition*
A0A8C1HM42	Ig-like domain-containing protein	1.78	Ig-like domain profile	ighv10-1	One-to-many	No	Immune recognition*
A0A8C1HPR1	Si:ch211-288g17.4	3.70	Not identified	si:ch211-288g17.4	One-to-many	Yes	Function uncertain; orthology-supported but poorly characterized
A0A8C0XYD7	Vitamin D-binding protein	3.91	Albumin domain profile	gc	One-to-many	Yes	Lipid/sterol transport and systemic carrier protein function**
A0A8C1F0F4	C-type lectin domain-containing protein	4.84	C-type lectin domain	Not identified	–	–	Carbohydrate recognition and innate immune-related processes*

Note. *domain-based prediction; **prediction supported by conserved orthology.
